# The Role of Epithelial Damage in the Pulmonary Immune Response

**DOI:** 10.3390/cells10102763

**Published:** 2021-10-15

**Authors:** Rachel Ann Burgoyne, Andrew John Fisher, Lee Anthony Borthwick

**Affiliations:** 1 Fibrosis Research Group, Biosciences Institute, Newcastle University, Newcastle upon Tyne NE2 4HH, UK; Rachel.Burgoyne@newcastle.ac.uk; 2 Regenerative Medicine, Stem Cells and Transplantation Theme, Translational and Clinical Research Institute, Newcastle University, Newcastle upon Tyne NE2 4HH, UK; A.J.Fisher@newcastle.ac.uk; 3 Institute of Transplantation, Newcastle upon Tyne Hospitals NHS Foundation Trust, Newcastle upon Tyne NE7 7DN, UK; 4 Fibrofind, Medical School, Newcastle University, Newcastle upon Tyne NE2 4HH, UK

**Keywords:** idiopathic pulmonary fibrosis (IPF), chronic obstructive pulmonary disease (COPD), coronavirus, COVID-19, epithelial damage, necrosis, necroptosis, damage-associated molecular patterns (DAMPs), senescence, lung

## Abstract

Pulmonary epithelial cells are widely considered to be the first line of defence in the lung and are responsible for coordinating the innate immune response to injury and subsequent repair. Consequently, epithelial cells communicate with multiple cell types including immune cells and fibroblasts to promote acute inflammation and normal wound healing in response to damage. However, aberrant epithelial cell death and damage are hallmarks of pulmonary disease, with necrotic cell death and cellular senescence contributing to disease pathogenesis in numerous respiratory diseases such as idiopathic pulmonary fibrosis (IPF), chronic obstructive pulmonary disease (COPD) and coronavirus disease (COVID)-19. In this review, we summarise the literature that demonstrates that epithelial damage plays a pivotal role in the dysregulation of the immune response leading to tissue destruction and abnormal remodelling in several chronic diseases. Specifically, we highlight the role of epithelial-derived damage-associated molecular patterns (DAMPs) and senescence in shaping the immune response and assess their contribution to inflammatory and fibrotic signalling pathways in the lung.

## 1. Introduction

The pulmonary epithelium is a complex, tightly regulated system required for effective respiratory function, which differs considerably depending on its location within the respiratory tract and displays diverse physiological roles. The various regions of the respiratory tract are comprised of multiple cell types, each encompassing an expansive network of cell–cell interactions and regulatory mechanisms that are required to maintain tissue homeostasis and mount an effective response to damage [1]. Specialised epithelial cell populations ranging from simple ciliated cuboidal cells to pseudostratified ciliated, columnar cells line the proximal airways, while the distal alveolar region is comprised of alveolar epithelial cells (AECs) [2]. AECs can be further classified into type I or type II pneumoctyes, which are phenotypically and functionally distinct. Type I pneumoctyes (AECI) make up approximately 90–95% of the alveolar surface, providing a specialised surface for gas-exchange [3], and type II pneumoctyes (AECII) produce and secrete pulmonary surfactant to prevent alveolar collapse and aid lung compliance [4]. Recent advances in single-cell RNA sequencing technology and lineage tracing analysis are drastically transforming our understanding of pulmonary cell heterogeneity, with comprehensive reviews by Davis et al. [2] and Hewitt et al. [5] providing detailed information on pulmonary cell composition and the identification of novel cell populations.

In the lung, epithelial cells are situated at the interface between the internal and external environment and exert many important functions to maintain tissue integrity, including mucociliary clearance, maintenance of tight junctions and epithelial adherence and secretion of antibacterial, antimicrobial and antiprotease molecules [6,7,8,9,10,11]. Due to their location, pulmonary epithelial cells are uniquely susceptible to injury and face substantial challenges to tissue integrity such as routine exposure to a range of exogenous stressors including, but not limited to, bacterial and viral insult, cigarette smoke, asbestos and airborne pollutants [12]. Additionally, epithelial damage may also arise in response to endogenous signals such as oxidative stress—triggering DNA damage and ATP depletion [13].

In response to injury, epithelial cells are required to orchestrate the immune response and mediate clearance of invading pathogens and dead/damaged cells to facilitate resolution of inflammation and restore tissue homeostasis [14]. However, in many chronic lung diseases this process becomes dysregulated, resulting in a persistent inflammatory response, pathological wound repair and loss of functional tissue architecture [15]. Epithelial damage is a common feature of many chronic pulmonary diseases, with activation of cell death cascades often underlying the disease pathogenesis. Interestingly, damage to epithelial cells in different regions of the respiratory epithelium has been shown to be involved in vastly different disease courses including IPF [16], COPD [17] and COVID-19 [18].

This review will focus on the role of pulmonary epithelial cells as key mediators of the immune response to damage as well as exploring their role in the pathogenesis of chronic lung diseases. We will review how epithelial cells drive inflammation and fibrosis through interactions with immune cells and fibroblasts and explore emerging concepts around the role of epithelial damage and cell death cascades in pulmonary disease.

## 2. Sensing Danger: PAMP and DAMP Signalling

As well as providing a mechanical barrier to prevent damage, airway epithelial cells also act to coordinate the immune response in the lung. Rapid recognition of both exogenous and endogenous stressors by epithelial cells is therefore essential to facilitate a timely response to damage [19]. Accordingly, epithelial cells express multiple pattern recognition receptors (PRRs) such as toll-like receptors (TLRs) and nod-like receptors (NLRs), which are able to sense pathogen-associated molecular patterns (PAMPs) and damage-associated molecular patterns (DAMPs) [20,21]. Diverse roles of PRR’s have been described in the literature, with different cell types expressing different receptors, resulting in activation of distinct signalling pathways. Of the PRRs, TLRs are the most extensively studied and have been identified both at the cell surface (e.g., TLR1-6, 10) and intracellularly (e.g., TLR3, TLR7-9) in multiple cell types [6]. Stimulation of PRRs via PAMPs/DAMPs is a critical component of the innate immune response to damage, causing activation of numerous proinflammatory pathways involved in the immune response to pathogens as well as contributing to disease pathogenesis of non-infectious lung diseases. For example, TLR4 and the receptor for advanced glycation end-products (RAGE) are widely expressed throughout the respiratory epithelium, where they have pivotal roles in triggering the proinflammatory response [22,23].

A wide array of molecules can act as PAMPs including bacterial DNA, cell wall components (e.g., lipopolysaccharide (LPS), peptidoglycan) and viral single and double stranded RNA [24,25]. Interactions between PAMPs and PRRs are key triggers of the immune response to invading pathogens and are recognised by PRRs on a wide range of cell types including epithelial cells, immune cells and fibroblasts [26]. Failure to clear pathogens and/or PAMPs from the microenvironment provides a continuing source of receptor activation, perpetuating the inflammatory response and resulting in damage to the lungs. Numerous pulmonary diseases are believed to have an infectious aetiology which drives inflammation and obstructs tissue function. For example, following lung transplantation, sustained colonisation of the allograft with *Pseudomonas aeruginosa* is strongly linked to development of bronchiolitis obliterans syndrome (BOS) [27], and exacerbations of COPD are frequently associated with recurrent viral and bacterial infection [28].

In addition to sensing danger arising from invading pathogens, it has become increasingly clear that PRRs can also detect and respond to molecules released from damaged and dying cells in the absence of microbial infection [29]. Collectively referred to as DAMPs, these molecules act as crucial danger signals or ‘alarmins’ which alert the immune system to tissue injury and elicit a ‘sterile’ inflammatory response in the absence of exogenous pathogens or infection [30]. Under normal conditions, DAMPs remain intracellular, where they exert diverse roles on homeostasis. However, in response to non-pathogenic tissue trauma, damaged or dying cells lose membrane integrity, resulting in the passive release of DAMPs into the extracellular space. These include nuclear and cytosolic proteins and nucleotides, such as high-mobility group box 1 (HMGB1), heat-shock proteins (HSPs), histones, galectin-3, interleukin (IL)-33 and IL-1α, amongst others (see Table 1) [31,32]. DAMPs play an important role in physiological wound healing by promoting re-establishment of cellular integrity and alerting neighbouring cells to danger. However, accumulating evidence suggests that DAMPs may actively contribute to several chronic inflammatory, fibrotic and autoimmune diseases [33,34,35]. Given that the respiratory epithelium is constantly subject to damage from endogenous and environmental stressors, the role of epithelial-derived DAMPs is of particular interest when considering the pathogenesis of pulmonary diseases.

## 3. Epithelial–Immune Cell Cross Talk

Interactions between epithelial cells and immune cells are essential to maintain tissue homeostasis, mount an effective immune response and promote resolution of epithelial injury. Acute inflammation is considered an integral component of a normal wound healing response to epithelial damage [111,112]. This is a highly synchronised and carefully regulated process involving the secretion of multiple cytokines and chemokines to stimulate recruitment and activation of inflammatory cells to the site of injury [113]. Reciprocal activation and regulation between epithelial cells and immune cells are therefore crucial and facilitated by both direct and indirect mechanisms. Like epithelial cells, innate immune cells express a broad range of PRRs and are consequently able to sense and respond to epithelial-derived stressors to shape the immune response. Persistent stimulation of PRRs on immune cells by epithelial-derived DAMPs can result in dysregulation of normal wound healing responses, causing a hyper inflammatory microenvironment, tissue destruction and dysregulation of normal wound healing [114]. Multiple immune cell types are reported to have altered signalling in pulmonary disease, effectively skewing the immune response to perpetuate inflammation and fibrosis.

One of the most widely investigated DAMPs is IL-33, a member of the Interleukin-1 superfamily of cytokines [46]. As well as operating as a classical cytokine, IL-33 can be released into the tissue microenvironment in response to damage and function as a key alarmin to trigger the immune response. Once in the extracellular space, IL-33 is cleaved to its modified form by neutrophil and mast cell proteases to drive a type 2 immune response characterised by the presence of regulatory T cells, eosinophils and cytokines such as IL-4, IL-5 and IL-13 [115,116]. To achieve this, IL-33 binds to ST2-expressing cells (including innate and adaptive immune cells) and activates the nuclear factor-κB (NF-κB) pathway to stimulate production of cytokines/chemokines and the degranulation of eosinophils, mast cells and basophils [115]. IL-33 can also promote the differentiation of macrophages towards an alternatively activated, pro-fibrotic M2 phenotype, causing upregulation of pro-fibrotic cytokines including monocyte chemoattractant protein (MCP-1), IL-6 and transforming growth factor (TGF)-β1 [117,118].

IL-33 is highly expressed in pulmonary epithelial cells and has been shown to exacerbate airway inflammation and tissue damage in respiratory disease [119]. For example, IL-33 and ST2 are both significantly increased in the plasma of COPD patients [120] and in the lungs of cigarette smoke-challenged mice [121], coinciding with increased infiltration of neutrophils and macrophages and expression of inflammatory cytokines/chemokines. IL-33 has been implicated as a driver of chronic airway inflammation and has been shown to induce upregulation of proinflammatory cytokines such as IL-6 and IL-8 in epithelial cells and peripheral blood mononuclear cells of COPD patients [122,123]. Work by Kearley et al. further demonstrated that cigarette smoke challenge in vivo caused upregulation of epithelial-derived IL-33 and modulation of ST2 expression to favour a T helper (Th)1-like proinflammatory immune cell response [124]. Importantly, IL-33 depletion successfully attenuated the proinflammatory phenotype, suggesting that IL-33 plays a critical role in cigarette smoke-induced airway inflammation [124]. Recently, IL-33 has emerged as a potential therapeutic target for the treatment of asthma and COPD, with a review by Donovan et al. highlighting key clinical evidence for targeting IL-33 in human respiratory disease [125].

Numerous studies have also demonstrated a pro-fibrotic role for IL-33 in driving excessive repair and remodelling pathways in pulmonary fibrosis. IL-33 has been reported to be upregulated in the bronchoalveolar lavage (BAL) and lung tissue of IPF patients [126] and in experimental models of disease [127], with accumulation of IL-33+ cells observed in bleomycin-challenged mice. Furthermore, full length IL-33 exacerbates inflammation and fibrogenesis in vivo, potentially through upregulation of TGF-β, as well as increasing expression of heat shock proteins including HSP70 [127]. Interestingly, ST2 depletion protects mice from developing bleomycin-induced lung fibrosis, suggesting that recruitment of ST2+ immune cells by IL-33 is an important factor driving fibrogenesis [128]. Similarly, other DAMPs such as HMGB1 and S100 proteins can shape the immune contribution to fibrosis and promote Th2 signalling. HMGB1, which has been shown to be upregulated in epithelial cells of pulmonary diseases including IPF [129] and COPD [130], can stimulate macrophages to increase production of IL-1β [131] and release of chemokines such as MCP-1 from epithelial cells [132]. MCP-1/chemokine receptor type 2 (CCR2) signalling is a key trigger in the initiation and progression of pulmonary fibrosis [133], suggesting epithelial-derived HMGB1 can contribute to the development of fibrosis through persistent secretion of MCP-1 in response to damage. Additionally, S100A8 and S100A9 are constitutively expressed by immune cells including monocytes and neutrophils and are released in response to cellular injury, where they can act as DAMPs to stimulate chemotaxis of other immune cells to the site of damage [134,135]. Infiltrating neutrophils and activated resident macrophages then increase expression of proinflammatory cytokines, reactive oxygen species, proteases and DAMPs in the tissue microenvironment, which in turn can drive damage to epithelial cells [136]. S100A8/A9 has also been proven to directly activate epithelial cells via stimulation of TLR4, resulting in increased secretion of several inflammatory cytokines including IL-6, MCP-1 and IL-8 [137]. These results highlight the ability of epithelial damage to shape the immune response through activation and recruitment of immune cells, which in turn can then stimulate further damage to the epithelium. If unresolved, persistent inflammatory activation can drastically alter cytokine signalling in the lung, causing a shift in the immune response, giving rise to fibrosis and impeding effective wound repair.

## 4. Epithelial–Fibroblast Cross Talk

Following injury, resident tissue fibroblasts proliferate and differentiate into myofibroblasts and begin secretion of extracellular matrix (ECM) and pro-fibrotic factors to promote edge contractility and facilitate wound closure [138]. Fibroblasts have long been considered as cells whose function was limited chiefly to restoration of tissue architecture. However, it is becoming increasingly apparent that fibroblasts play other critical roles in wound healing and that interactions between the pulmonary epithelium and fibroblasts play a major role in tissue homeostasis, as well as in the initiation and progression of several chronic lung diseases [139]. For example, fibroblasts have been identified as having a critical role in regulating the switch from acute to chronic inflammation through modulation of immune cell function [140]. Indeed, evidence suggests that dysfunctional fibroblasts can induce a persistent inflammatory state in the cellular microenvironment through secretion of proinflammatory cytokines, driving accumulation of immune cells and impeding wound repair [141]. Suwara et al. successfully demonstrated that pulmonary fibroblasts challenged with conditioned media from damaged/dying epithelial cells undergo a phenotypic switch to a proinflammatory state, characterised by the secretion of chemokines involved in the recruitment of neutrophils, monocytes and T cells. This phenotypic switch was driven exclusively via IL-1α released from damaged epithelial cells engaging interleukin 1 receptor type 1 (IL-1R1) on fibroblasts, and this effect could be attenuated using an IL-1α neutralising antibody or IL-1R1 antagonist, suggesting that IL-1α is a key epithelial-derived factor driving a proinflammatory phenotype in human fibroblasts [141]. These findings were confirmed in subsequent in vitro studies, whereby airway epithelial cells isolated from COPD patients were shown to directly influence the downstream activity of lung fibroblasts and upregulate proinflammatory cytokine secretion through the release of IL-1α [142]. Additionally, cigarette smoke exposure was found to enhance IL-1α release and exacerbate the proinflammatory phenotype of fibroblasts, with this effect enhanced in epithelial cells isolated from severe COPD patients [142]. Taken together, these data suggest that epithelial damage and release of intracellular alarmins can initiate aberrant crosstalk between epithelial cells and fibroblasts to drive inflammation and alter wound repair signalling. Single-cell sequencing of mouse and human lungs have further identified subpopulations of pulmonary fibroblasts unique to fibrotic lungs that can adopt a pro-fibrotic phenotype. Functionally, these cells exhibit enhanced migratory functions and localise to regions of fibrotic tissue, suggesting an important role in driving pulmonary fibrosis [143]. Despite these observations, the role of the damaged epithelium in fibrosis and its downstream effects on fibroblasts remain poorly understood. Defining how epithelial cell damage stimulates the fibrotic response and identifying the molecular crosstalk mechanisms between damaged/dying epithelial cells and fibroblasts may be instrumental to improve our knowledge of the pathophysiology of pulmonary fibrosis and enable identification of potential biomarkers or therapeutic targets to attenuate crosstalk.

## 5. Epithelial Damage and COPD

**Figure 1:**
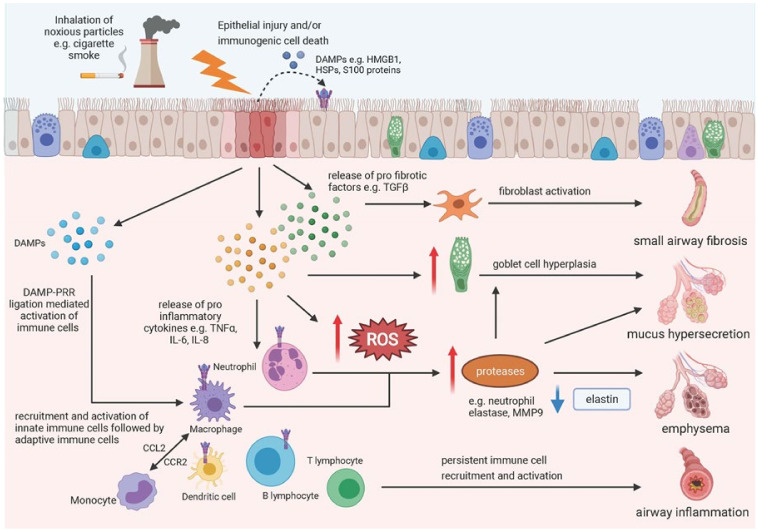
Schematic diagram of downstream effect of epithelial damage in COPD. Inhalation of noxious particles (e.g., cigarette smoke) is a major risk factor in the development of COPD, causing epithelial injury, immunogenic cell death and the release of DAMPs and pro inflammatory/fibrotic chemokines/cytokines into the extracellular space. DAMPs can activate pattern recognition receptors (PRRs) on neighbouring epithelial cells and immune cells, directly stimulating the release of chemokines and cytokines to activate the immune system. Several mechanisms have been implicated in the development of COPD, including the influx of inflammatory cells, proteolytic imbalance and oxidative stress, resulting in airway inflammation, emphysema, mucus hypersecretion and small airway fibrosis. Created using Biorender.com (accessed on 5 October 2021).

Chronic obstructive pulmonary disease (COPD) is a common respiratory disease characterised by progressive airflow obstruction, an accelerated decline in lung function and a combination of epithelial damage (bronchitis) and destruction of the lung parenchyma (emphysema) [144]. COPD is characterised by an uncontrolled inflammatory response, causing sustained airway inflammation leading to dysregulated tissue repair and airway remodelling [145]. Chronic exposure to noxious particles is a major risk factor in the development of COPD, with cigarette smoke exposure widely regarded as one of the most prevalent stressors underpinning disease pathogenesis [146]. Though several mechanisms have been implicated in the development of the disease, including the influx of inflammatory cells, proteolytic imbalance and oxidative stress, the pathways driving progression remain ill-defined [147]. One hypothesis in the literature suggests that cigarette smoke induces airway epithelial injury, driving immunogenic cell death, DAMP release and subsequent triggering of the immune response (see Figure 1). This is supported by several studies both in vitro and in vivo which have successfully demonstrated that epithelial cells exposed to cigarette smoke (CS) undergo a switch from apoptotic to necrotic cell death [148,149], coinciding with the release of DAMPs, which stimulate the innate immune response [150,151,152]. In 2016, Pouwels et al. showed for the first time that expression of DAMPs and PRRs are dysregulated in COPD patients, with enrichment of genes encoding galectin-3, TLR2, TLR4, and S100A9 identified in bronchial brushings [153]. Interestingly, the use of in vivo mouse models further indicated that enhanced responsiveness to DAMPs may increase susceptibility to cigarette smoke-induced airway inflammation. A follow up study identified *CFLAR*, a gene encoding c-FLIP (cell death regulator), as a susceptibility gene for cigarette smoke-induced airway inflammation and confirmed that *CLFAR* expression is decreased in response to cigarette smoke extract (CSE). Moreover, downregulation of *CFLAR* in pulmonary epithelial cells increased necrotic cell death and release of DAMPs in response to CSE, enhancing the inflammatory response [150].

Unlike apoptosis, necrotic death is viewed as an uncontrolled, highly immunogenic form of cell death resulting in the loss of plasma membrane integrity and the passive release of cellular contents into the extracellular space. Numerous DAMPs have now been found to be elevated in the serum or lung fluid of COPD patients, including S100 proteins, heat shock proteins and HMGB1, likely contributing to the persistent inflammatory microenvironment seen in disease [41,154,155,156]. For example, HMGB1 is a potent inflammatory mediator that triggers the immune response through stimulation of PRRs including TLR2, TLR4 and RAGE, resulting in the nuclear translocation of NF-κB and production of inflammatory cytokines and chemokines [42,157]. The role of DAMPs in driving airway inflammation in COPD is supported by clinical observations, where elevated levels of HMGB1 in the sputum of COPD patients is inversely correlated to forced expiratory volume (FEV1) [41]. Notably, HMGB1, S100A8 and IL-37 (all RAGE ligands) have also been reported to increase during COPD exacerbations [154], with *ager* (gene encoding RAGE) identified as a susceptibility gene in COPD [158].

**Figure 2:**
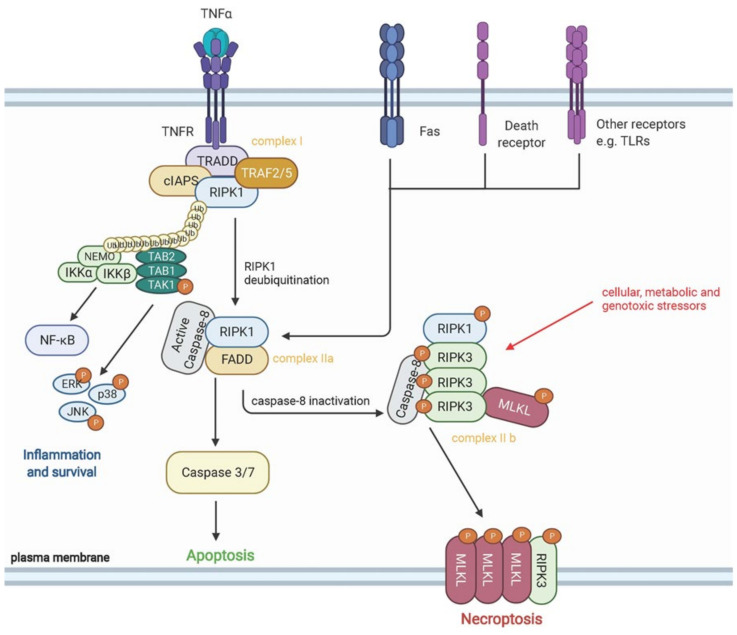
Schematic diagram of pathways involved in necroptosis. Necroptosis can be triggered downstream of death domain receptors (e.g., TNFR and Fas), toll-like receptors (e.g., TLR-4 and TLR-3) or in response to cellular, metabolic and genotoxic stressors. Upon activation of receptors, recruitment of adaptor proteins including TRADD, TRAF2/5, RIPK1, cIAPs and other molecules occurs to form complex I. Polyubiquitination of RIPK1 by cIAPs prevents RIPK1 function and activates NF-kB, leading to expression of proinflammatory cytokines and cell survival. Conversely, RIPK1 deubiquitination by CYLD leads to the formation of complex IIa, with Caspase-8 activation preventing activation of RIPK1 and necroptosis. Inactivation of Caspase-8 in complex IIa causes phosphorylation and activation of RIPK1, RIPK3 and MLKL during the assembly of complex 11b—also known as the necrosome. Phospho-MLKL oligomers then translocate to the plasma membrane and form large pores, leading to necroptotic cell death by facilitating ion influx, cell swelling and membrane lysis, followed by the passive release of intracellular molecules into the tissue microenvironment. Created using Biorender.com.

It has long been considered that the main distinction between apoptotic and necrotic cell death was the ability, or inability in the case of necrosis, to programme molecular events in response to damaging stimuli. However, within the last decade it has become increasingly clear that in some settings, necrotic cell death can be modulated by distinctive molecular pathways [159,160]. A novel form of necrosis—termed necroptosis—has now been described, which is highly regulated and dependent on two serine–threonine kinases: receptor-interacting protein kinase-1 (RIPK1) and receptor-interacting protein kinase-3 (RIPK3). Necroptosis arises in response to stimulation of death receptors, TLRs or interferon signals and is dependent on RIPK3 phosphorylation and its subsequent complexing with RIPK1 to form the necrosome in the absence of activated caspases [160]. Phosphorylated RIPK3 acts downstream through phosphorylation of mixed lineage kinase domain like pseudokinase (p-MLKL), which functions as the executioner of necroptosis (see Figure 2) [161]. Like necrosis, necroptosis is an inflammatory form of cell death resulting in the release of DAMPs into the extracellular space. DAMPs released via necroptosis have been implicated in the pathogenesis of several diseases, with recent studies identifying potential roles in acute kidney injury [162], osteoarthritis [163] and cardiovascular disease [164].

Accumulating data now indicates a potentially key role of airway epithelial cell necroptosis in the pathogenesis of COPD, with evidence of increased necroptotic markers in both human patients and experimental models of COPD correlated to disease severity. For example, Chen et al. found that HMGB1 and p-MLKL were markedly upregulated in CS-induced mouse models of emphysema, CSE-treated 3D lung organoid models and in the lungs of COPD patients [165]. Additionally, CS-induced necroptotic cell death triggered enhanced but slower clearance of damaged cells by macrophages, leading to increased levels of proinflammatory cytokine tumour necrosis factor (TNF)-α and IL-6 in the lung. Furthermore, they showed that pharmacological or genetic inhibition of RIPK3 (using RIPK3 inhibitor GSK’872) in vivo and p-MLKL (using CRISPR/Cas9 technology) in vitro attenuated CSE-induced cell death and suppressed neutrophilic airway inflammation [165]. These findings were subsequently confirmed by Lu et al., who similarly found increased necroptotic markers in experimental models of COPD, with depletion of RIPK3 and p-MLKL preventing airway inflammation and tissue remodelling following cigarette smoke exposure [166]. These data suggest that necroptotic cell death is a key trigger of airway inflammation in response to cigarette smoke exposure in COPD patients, which potentiates tissue remodelling and the development of emphysema. However, other studies have provided evidence of alternative cell death pathways involved in COPD pathogenesis including ferroptosis (an iron-dependent regulated form of cell death) [167] and cigarette smoke-induced mitochondrial autophagy [168,169]. This suggests that modulating cell death pathways or selectively targeting epithelial-derived DAMPs could have beneficial effects in disease pathogenesis. Further work is therefore needed to unpick the molecular mechanisms by which damaged epithelial cells might interact with other cell types downstream to drive aberrant wound repair and inflammation.

## 6. Epithelial Damage and IPF

**Figure 3:**
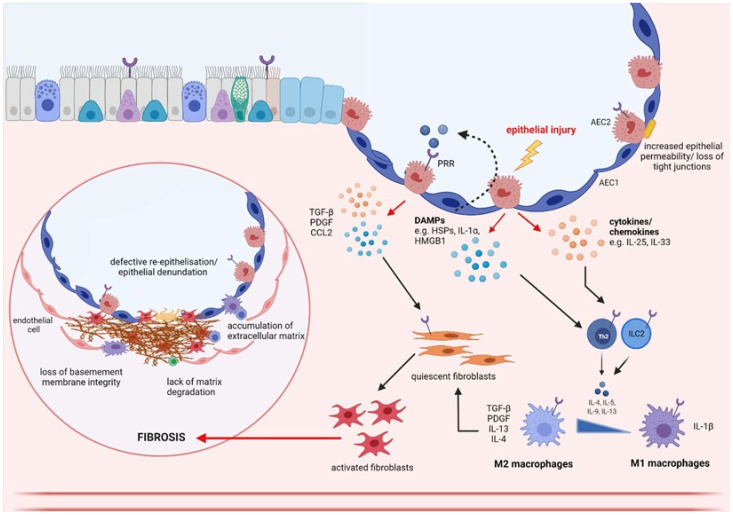
Schematic diagram of downstream effects of epithelial damage in IPF. Upon injury, epithelial cells release chemokine/cytokines and DAMPs (e.g., high-mobility group box-1 (HMGB1), heat shock proteins (HSPs) and interleukin (IL)-1α) into the extracellular space. DAMPs can activate pattern recognition receptors (PRRs) on neighbouring epithelial cells and immune cells, directly stimulating the release of profibrotic cytokines including tumour growth factor (TGF)-β, PDGF and CCL2, which are involved in the activation of fibroblasts. Epithelial cells also secrete proinflammatory cytokines which recruit and activate innate immune cells (e.g., neutrophils, macrophages and dendritic cells), as well as adaptive immune cells (e.g., T lymphocytes and B lymphocytes), which further secrete pro fibrotic factors including IL-33, IL-4, IL-5, IL-13. For example, IL-33 promotes the differentiation of macrophages towards to a pro-fibrotic M2 phenotype, causing upregulation of pro-fibrotic cytokines including monocyte chemoattractant protein (MCP-1), IL-6 and TGF-β. Once activated, fibroblasts begin secretion of extracellular matrix (ECM) and pro-fibrotic factors to promote edge contractility and facilitate wound closure. Fibrosis is thought to occur in response to persistent epithelial damage leading to continued proliferation and migration of myofibroblasts, deposition of extracellular matrix (ECM) and recruitment of pro-inflammatory and pro-fibrotic markers with detrimental effects. Created using Biorender.com.

Idiopathic pulmonary fibrosis (IPF) is a chronic interstitial lung disease of unknown aetiology characterised by the aberrant deposition ofECM in multifocal regions of the lung parenchyma, alongside seemingly normal regions of tissue [170]. Though IPF was first thought to be an inflammatory-driven disease, strong experimental data and the failure of anti-inflammatory and immunosuppressive drugs in clinic have since challenged this theory [171]. The current paradigm now suggests that IPF is an epithelial-driven disease whereby repeated and persistent epithelial injury is a key initiating event triggering fibroblast activation and ECM deposition (see Figure 3) [16].

Although DAMPs primarily function to stimulate the immune response, several studies have highlighted a possible role for them in fibrogenesis in multiple organs [172,173]. In the context of the lung, DAMPs including HMGB1, S100 proteins, uric acid and extracellular ATP are all significantly increased in BAL fluid of IPF patients compared with healthy controls [174,175,176,177,178]. Though information on the cellular origin of these DAMPs in humans is limited, histological assessment of IPF samples found that HMGB1 was predominantly expressed in the nuclei of infiltrating inflammatory cells and epithelial cells in fibrotic IPF lesions [129]. Likewise, HMGB1 was found to be upregulated primarily in bronchiolar and alveolar epithelial cells in bleomycin-induced fibrotic mouse models [129]. Several studies have now demonstrated a potentially key role for epithelial-derived DAMPs in the pathogenesis of pulmonary fibrosis, with release of alarmins from epithelial cells enhancing the fibrotic response in multiple systems. For instance, extracellular ATP has been found to cause upregulation of TGF-β1, collagen and fibronectin in pulmonary fibroblasts [179]. Interestingly, depletion of HMGB1 using neutralising antibodies [129] and ethyl pyruvate (a HMGB1 inhibitor) [180] in bleomycin-challenged mice successfully attenuated the fibrotic response, suggesting a key role in fibrogenesis. Though the involvement of HMGB1 in fibrosis is incompletely understood, some studies suggest that activation of fibroblasts and production of IL-1β are key drivers in disease progression. Indeed, it has been shown that HMGB1 released from damaged epithelial cells in vitro causes upregulation of IL-1β, which in turn activates TGF-β1 to facilitate alveolar epithelial cell repair [181]. Li et al. provided the first evidence that HMGB1 can mediate epithelial-to-mesenchymal transition in both human and rat airway epithelial cells through activation of TGF-β1/Smad2/3 signalling pathways [182]. Wang et al. subsequently showed that HMGB1-induced TGF-β1 release precedes the upregulation of α-smooth muscle actin (SMA) and collagen I in pulmonary fibroblasts, indicating that TGF-β1 is required for the differentiation of human lung fibroblasts to myofibroblasts in response to HMGB1 [183]. Collectively, these studies provide novel evidence that HMGB1 released from damaged epithelial cells might contribute to the development of fibrosis through persistent upregulation of TGF-β1, causing fibroblast activation, differentiation and deposition of ECM.

More recently, increased expression of necroptotic markers including RIPK3 and p-MLKL have been observed in IPF patient samples, particularly within alveolar epithelial cells [184]. Consistent with these findings, murine alveolar epithelial cells challenged with bleomycin were found to upregulate expression of RIPK3, p-MLKL and release the alarmins HMGB1 and IL-1β. Furthermore, RIPK3 knock-out mouse models confirmed attenuated induction of cell death, inflammation and fibrosis in response to bleomycin challenge—suggesting that necroptosis contributes to development of fibrosis. This finding was further supported by treatment of wild type mice with Necrostatin-1 (a RIPK1 inhibitor used to prevent necroptosis) after bleomycin challenge, which resulted in a marked reduction of MLKL phosphorylation and downstream inflammation and fibrosis [184]. To date, few studies have investigated the role of necroptosis in IPF. However, as new information comes to light, it is becoming increasingly clear that necrotic cell death and the subsequent release of DAMPs play key roles in orchestrating tissue injury and repair mechanisms. Targeting the necroptotic pathway and/or the DAMPs released from necrotic cells may therefore have therapeutic benefits in pulmonary disease. Several inhibitors of the necroptosis pathway are currently available for use in experimental settings and have provided promising results in vitro and in mouse models of disease. These inhibitors include RIPK1-specfic necrostatins, RIPK3-specific compounds (e.g., GSK′872, GSK′843 and Dabrafenib), RIPK1–RIPK3 dual inhibitors (e.g., GSK′074) and MLKL inhibitors (e.g., necrosulfonamide) [185,186,187,188]. However, these compounds require further development and clinical testing to assess their efficacy in humans.

## 7. Epithelial Damage and COVID-19

**Figure 4:**
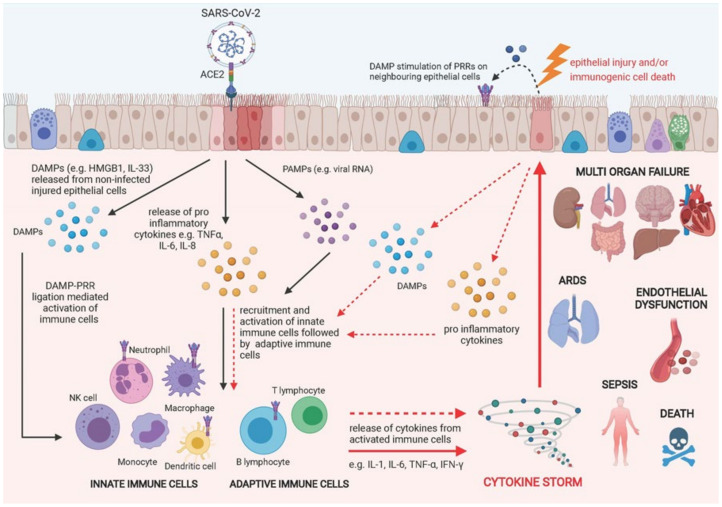
Schematic diagram of downstream effects of epithelial damage in COVID-19. COVID-19 infection occurs when the severe acute respiratory syndrome coronavirus 2 (SARS-CoV-2) spike protein binds to surface receptor angiotensin-converting enzyme 2 (ACE2) on pulmonary epithelial cells, stimulating the release of PAMPs, DAMPs and cytokines/chemokines into the cellular microenvironment and causing the recruitment and activation of innate immune cells followed by adaptive immune cells to the site of damage. In most circumstances, the immune response is capable of resolving infection and restoring tissue homeostasis. However, this process can become dysregulated, resulting in a hyper inflammatory response termed a ‘cytokine storm’, which can cause further damage to the pulmonary epithelium in a positive feedback loop. Damaged epithelial cells stimulate the release of more pro-inflammatory chemokines/cytokines and DAMPs, exacerbating epithelial cell damage and death. Cytokine storm syndrome is considered to be one of the major causes of acute respiratory distress syndrome (ARDS), endothelial dysfunction, sepsis and multiple-organ dysfunction in humans, causing a rapid decline in lung function and ultimately death. Created using Biorender.com.

Coronavirus disease 2019 (COVID-19) is a rapidly emerging respiratory disease ranging from asymptomatic infection to rapidly progressing interstitial pneumonia, respiratory failure and mortality [189]. Infection occurs when the severe acute respiratory syndrome coronavirus 2 (SARS-CoV-2) spike protein directly binds to the host cell surface receptor angiotensin-converting enzyme 2 (ACE2) to facilitate viral entry and replication [190,191]. ACE2 is highly expressed on pulmonary epithelial cells—particularly alveolar epithelial cells—with Zhao et al. reporting that 83% of ACE2+ cells are type 2 pneumocytes [192]. Upon infection, the immune system is quickly activated to mediate recruitment of innate immune cells to respond to infection, coupled with the release of cytokines to prime the adaptive immune response to aid tissue repair. In the majority of cases, this process is capable of resolving infection and restoring tissue homeostasis. However, in some cases, infection can trigger a hyper inflammatory response in the pulmonary microenvironment termed a ‘cytokine storm’ with numerous detrimental effects (see Figure 4) [193]. Cytokine storm syndrome is considered to be one of the major causes of acute respiratory distress syndrome (ARDS) and multiple-organ dysfunction in humans, causing a rapid decline in lung function and ultimately death [194,195].

Epithelial damage and death have been highlighted as key drivers of disease severity within the current literature. Indeed, significant upregulation of genes encoding cell death receptors (e.g., *FAS* and *TNFRS1A*) and pro-apoptotic proteins (e.g., caspase 8, caspase 3 and cytochrome c), have been identified in ciliated and secretory epithelial cells isolated from patients with moderate–severe COVID-19 [196]. Recent observations have found upregulated serum levels of the DAMP cytokine IL-33 in more severe cases of COVID-19, which could be attributed to epithelial damage causing increasing interactions between the airway epithelium and inflammatory immune cells [197]. SARS-CoV-2-derived papain like protease has been proven to be a potent inducer of IL-33 in pulmonary epithelial cells [198], with significant upregulation of IL-33 occurring in SARS-CoV-2 infected epithelial cells in vitro [199]. Furthermore, Chua et al. demonstrated that severe COVID-19 cases were associated with hyper-inflammatory macrophages and cytotoxic T cells, suggesting a direct correlation between the degree of epithelial–immune cell interactions and disease severity [196]. One possible explanation is that the heightened pro-inflammatory environment within the lung exacerbates epithelial cell death, which in turn stimulates the release of more inflammatory cytokines and chemokines in a positive feedback loop. Consistent with this hypothesis, proteomic analysis of plasma isolated from COVID-19 patients found upregulated expression of a number of severity-associated intracellular (e.g., RAGE) and proinflammatory proteins (e.g., IL-33 receptor, IL-1RL1) when compared with mild cases [200]. S100A8 is also significantly upregulated in COVID-19 patients and mouse models of SARS-CoV-2-infection, resulting in a dysregulated immune response and activation of a population of aberrant neutrophils [201,202,203].

Work by Filbin et al. suggests immunogenic epithelial cell death is a key feature of severe disease, with upregulation of multiple proteins in the plasma of COVID-19 patients showing signals for enrichment of HMGB1 signalling and the necroptotic pathway, in line with disease severity [200]. These findings were supported by Chen et al. who confirmed that HMGB1 was elevated in the serum of severe COVID-19 patients and found that HMGB1 induced the upregulation of ACE2 in epithelial cells through interactions with the DAMP receptor RAGE [204]. Similarly, Henry et al. established that COVID-19 is associated with higher rates of cell death, with necrotic cell death appearing to be the main driver of hospitalisation, whereas apoptosis and necrosis appear to drive intensive care admission [205].

Preliminary data therefore suggests an involvement of multiple pathways of epithelial cell death in severe COVID-19 cases, with evidence of both apoptotic and necrotic cell death in the literature. Notably, Li et al. postulated that epithelial cell death in response to SARS-CoV-2 infection was modulated through activation of caspase-8, a master regulator of cell death pathways [206]. In vitro experimentation confirmed that SARS-CoV-2 infection induced caspase-8 activation, epithelial apoptosis and processing of IL-1β to its active form. Further analysis found that the necroptotic pathway was required to secrete processed IL-1β and drive the inflammatory response. As caspase-8 is known to inhibit necroptosis [207], it is likely that SARS-CoV-2 induced a level of cleaved caspase-8, which would allow processing of IL-1β alongside necroptosis activation. This proposed dual mode of cell death was confirmed histologically in lung samples of fatal COVID-19 patients and infected mouse models where significant infiltration of inflammatory immune cells and interstitial fibrosis was observed, alongside prominent areas of apoptotic and necrotic cell debris [206]. However, depending on the degree of activation, viral-induced epithelial cell death can also have beneficial anti-viral effects to limit viral replication and resolve tissue injury [208], highlighting the complex balance between cell death cascades, their ability to shape the immune response and how aberrations in cell death signalling can have detrimental effects on tissue function.

Based on these data, there is a growing body of evidence which suggests that the cytokine storm seen in severe COVID-19 cases is consistent with dysregulated cell death signalling pathways and a hyper inflammatory immune response mediated by increased DAMP signalling in the lung. Consequently, DAMPs/necroptotic markers could prove to be a valuable prognostic biomarker to monitor and evaluate disease progression of SARS-CoV-2 infection. Moreover, due to their involvement in severe disease, future work should seek to consider DAMPs and the necrotic pathway as possible therapeutic targets.

## 8. Epithelial Damage and Senescence

**Figure 5:**
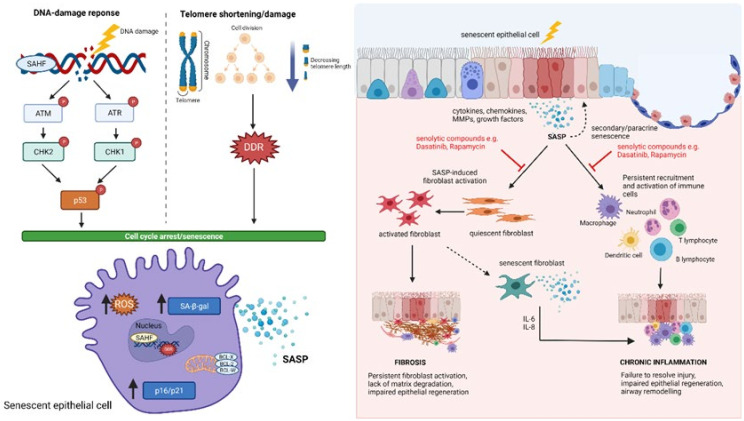
Schematic diagram of epithelial senescence in the lung. Cellular senescence can be triggered in response to different stimuli, including telomere shortening/damage and engagement of the DNA-damage response (DDR). Senescent cells upregulate expression of senescence-associated heterochromatin foci (SAHF), senescence-associated β-galactosidase activity (SA-β-gal), p16 and p21 and secrete a multitude of chemokines, cytokines, proteases, and growth factors known as the senescent-associated secretory phenotype (SASP). Senescent epithelial cells exert diverse roles in the lung due to the heterogeneity of SASP factors, including activation of fibroblasts and immune cells to drive fibrosis and inflammation, respectively. Senescent epithelial cells can also induce senescence in neighbouring cells, reinforcing growth arrest, impairing epithelial regeneration and efficient lung function. Senescent fibroblasts further impair lung function and repair of epithelial cells, suggesting bi-directionality and positive feedback mechanisms driving epithelial damage and fibroblast activation. Senolytic compounds including Dasatinib and Rapamycin can successfully attenuate fibroblast activation and immune cell recruitment in the lung, making modulation of senescence an attractive therapeutic target for several chronic diseases. Created using Biorender.com.

Senescence is a complex, multifaceted process resulting in permanent loss of the proliferative ability of cells. First described over 60 years ago, Hayflick and Moorhead observed that human foetal fibroblasts in culture could only divide a finite number of times before undergoing permanent cell cycle arrest [209]. This phenomenon, termed replicative senescence, has since been attributed to the gradual telomere shortening associated with extensive proliferation, resulting in engagement of the DNA damage response (DDR) and cells entering a senescent state [210]. Alongside telomere dysfunction, it has been shown that many stimuli can elicit cellular senescence, prematurely including DNA damage, ionizing radiation, oxidative stress, and cytotoxic therapies (stress-induced senescence) [211]. Unlike apoptotic and necrotic cells, senescent cells remain metabolically active and can affect the activity of themselves and neighbouring cells via secretion of a multitude of chemokines, cytokines, proteases, and growth factors known as the senescent-associated secretory phenotype (SASP) [212] (see Figure 5). Persistent and sustained exposure to the SASP has been shown to drive age-associated tissue dysfunction and, in some instances, results in a proinflammatory milieu known as ‘inflammaging’ [213,214]. Cellular senescence is now widely considered an important driving mechanism in several chronic lung diseases including COPD [215], IPF [216,217] and COVID-19 [218,219].

Emerging evidence suggests that senescence may be a key contributing factor to IPF pathophysiology, with numerous senescence biomarkers, including senescence-associated β-galactosidase activity (SA-β-gal), p16 and p21, identified in fibroblasts and epithelial cells of IPF patients [220,221]. While markers of senescence have been widely reported in IPF, the origin of these senescent cells and their downstream effects remain largely unclear. Several studies have confirmed that senescent cells in IPF patient samples are predominantly epithelial cells, which are spatially located proximal to fibroblastic foci, suggesting an active involvement in fibrogenesis [131,222,223]. Consistent with this, data from a recent pilot study found that depletion of senescent cells in IPF patients using the senolytic compound Dasatinib conferred significant clinical benefit and alleviated physical dysfunction [224]. Furthermore, in vivo studies have demonstrated that genetic and pharmacological ablation of senescent cells can successfully attenuate fibrosis and restore tissue function in experimental models of pulmonary fibrosis [225,226]. Under normal conditions, maintenance of the alveolar epithelium is achieved through the proliferation of AECII cells to restore function in response to damage. However, extensive evidence shows that this process is defective in IPF patients, resulting in pronounced areas of denundation (depletion of AECs) in fibrotic regions of tissue [227], coupled with abnormal activation of senescence pathways [228]. Genomic analysis has identified several genetic mutations associated with an increased risk of developing IPF, including telomerase reverse transcriptase (*TERT*), telomerase RNA component (*TERC*) and the regulator of telomere elongation helicase 1 (*RTEL1*)—all of which are linked to premature senescence and a reduced regenerative capacity of type II pneumocytes [229].

More recently, the crosstalk between senescent epithelial cells and fibroblasts has begun to be explored, with one study showing that co-culture of pulmonary fibroblasts with senescent epithelial cells resulted in increased activation of fibroblasts and expressed increased levels of α-SMA, collagen I and vimentin [131]. These data suggest that senescent epithelial cells can induce activation of fibroblasts downstream to drive fibrogenesis. Indeed, inhibition of epithelial senescence with rapamycin (mTOR inhibitor) effectively suppressed fibroblast activation and limited development of fibrosis [131]. TGF-β1-induced senescent epithelial cells have also been reported to trigger differentiation of fibroblasts into myofibroblasts via secretion of IL-1β [222]. Conversely, co-culture of alveolar epithelial cells with senescent fibroblasts was found to impair the function and repair of epithelial cells, suggesting bi-directionality and positive feedback mechanisms driving epithelial damage and fibroblast activation [230]. Of note, Orjalo et al. demonstrated that pulmonary fibroblasts increase the production and secretion of IL-1α in response to senescence-inducing stimuli. Moreover, they showed that IL-1α was an essential positive regulator of IL-6 and IL-8 secretion and that senescent fibroblasts could act in an autocrine manner to drive inflammation and reinforce senescent growth arrest in themselves and neighbouring fibroblasts [231].

Interest in the role of cellular senescence in COPD is also increasing, with multiple senescent biomarkers and SASP components reportedly driving inflammation and aberrant tissue remodelling in COPD patients [232,233]. Importantly, several of these biomarkers are significantly elevated in the epithelial cells of COPD patients, indicating a key role of epithelial senescence driving disease pathogenesis [234,235]. Cigarette smoke is a major contributor to COPD progression, containing reactive oxygen species which promote inflammation, oxidative stress and induction of the DDR in pulmonary epithelial cells [236]. Epithelial injury can activate cellular senescence, as demonstrated by Cottage et al. who found that CS-induced DNA damage triggered activation of the p16 pathway [234]. In this study, CS-challenged wild type mice exhibited reduced pulmonary function, development of emphysema and increased alveolar epithelial cell senescence. Alongside this, SASP molecules such as TGF-β1, IL-33 and matrix metalloproteinase (MMP)-12 were all upregulated in response to cigarette smoke exposure. Genetic depletion of p16 in transgenic mice had a protective effect on this phenotype and appeared to attenuate induction of proteolytic enzymes, inflammatory cytokines and increase proliferation of alveolar progenitor cells [234]. However, this was in contrast to a recent study by Sundar et al. who reported that deletion of p16 had no beneficial effect in a chronic model of CS-induced COPD [237]. Pulmonary fibroblasts isolated from COPD patients have also been shown to secrete higher levels of a number of SASP proteins compared with matched controls, many of which have been implicated in chronic inflammation [215]. However, it should be noted that is it currently unclear if the senescence found in COPD is driven by acute or chronic exposure to damage, which will affect the associated SASP profiles.

Interrogation of the pathways underpinning cellular senescence has unveiled PI3K (phosphoinositide-3-kinase)–mTOR (mammalian target of rapamycin) signalling as a potential mechanism driving the senescent phenotype in COPD patients. mTOR is a serine–threonine protein kinase involved in the regulation of cell growth, metabolism and survival, that is activated by oxidative stress [238]. Data from Houssaini et al. supports this theory, showing that activation of mTORC1/2 occurs concomitantly with markers of senescence in alveolar epithelial cells isolated from COPD patients [239]. Furthermore, rapamycin (mTOR inhibitor) successfully decreases mTOR activity and induction of cellular senescence in COPD-derived epithelial cells and mouse models of emphysema. Similarly, Cheng et al. found that administration of metformin (an AMPK activator which inhibits mTOR) successfully diminished inflammation, alveolar destruction and cellular senescence in CS-induced COPD [235].

The link between cellular senescence and ageing has been well described, with senescent cells found to accumulate in numerous age-related diseases. Consequently, it has been suggested that cellular senescence may also contribute to the increased severity of COVID-19 infection in the elderly, though the underlying mechanisms remain unclear [218,240,241]. Camell et al. demonstrated that senescent cells increase the inflammatory response to β-coronavirus mouse hepatitis virus (MHV) infection (a virus in the same family as SARS-CoV-2) in aged mouse models and that treatment with the senolytic compound fisetin could reduce adverse outcomes and increase levels of antiviral antibodies [242]. Moreover, they showed that senescent human kidney endothelial cells can act in a paracrine manner to suppress viral defence mechanisms and facilitate increased expression of viral entry proteins in non-senescent cells. This was coupled with a shift in the SASP to a more inflammatory, pro-fibrotic phenotype, with increased expression of IL-1α and IL-1β, which could then exacerbate the inflammatory response and drive senescence in neighbouring cells. However, data in humans is limited and further work is required to unpick the precise role of senescence in COVID-19 patients.

These data suggest that cellular senescence contributes significantly to the pathophysiology of several chronic lung diseases, making the modulation of senescence an attractive therapeutic target. Though more work is required to understand the precise mechanisms of senescent cell signalling, it is encouraging to see efficacy of senolytic drugs in a wide range of lung pathologies. Further mechanistic work is necessary to delineate the contribution of senescent cells to the development of pulmonary inflammation and fibrosis and to confirm the role of senescent cell types in respiratory disease.

## 9. Conclusions

A review of the literature highlights an increasing appreciation for the role of epithelial damage and epithelial-derived DAMPs as key drivers underlying disease pathogenesis of several inflammatory and fibrotic pulmonary diseases. Moreover, complex crosstalk between damaged/senescent epithelial cells with multiple cell types including immune cells and fibroblasts has been shown to shape the immune response to injury, driving aberrant wound repair and impeding tissue function. However, cross regulation between necroptosis and other modes of cell death increases the complexity of these pathways, and this is further complicated by the ability of different DAMPs to activate different signalling pathways. The heterogeneity of SASP components and their downstream effects on tissue function also obscures our understanding of the role of senescent cells in human disease, warranting further research. Though preliminary data targeting DAMPs/necroptotic markers in disease and efficacy of senolytic compounds in early studies is promising, further investigation is required to elucidate the mechanisms underpinning the cellular crosstalk networks which are triggered in response to damage of the epithelial compartment to drive disease progression. This in turn will illuminate potential targets for future therapeutic strategies to dampen the inflammatory and fibrotic response and promote physiological wound repair.

## Figures and Tables

**Table 1:** Table of well-characterised DAMPs, their receptors, and evidence in disease.

DAMP Ligand	Known Receptors	Evidence in Disease	Reference
Intracellular-Nuclear DAMPs			
HMGB1	TLR2, TLR4, TLR9, RAGE, CXCR4	Ischaemia reperfusion injury, sepsis, acute lung injury, chronic inflammatory and autoimmune diseases, viral infection, cancer	[34,36,37,38,39,40,41,42]
HMGN1	TLR4	Cancer	[43,44,45]
IL-1α	TLR2, TLR4, TLR7, TLR8, TLR9, IL-1R	Chronic inflammatory disease, sepsis, cancer	[46,47,48,49]
IL-33	ST2	Asthma, atopic dermatitis, anaphylaxis, allergic inflammation, fibrosis	[46,47,48,50,51,52]
DNA	TLR9, AIM2	Kidney disease, inflammation, cancer	[53,54,55,56,57,58]
RNA	TLR3, TLR7, TLR8, MDA5	Autoimmune disease, sepsis, acute lung injury, liver injury	[34,56,59,60,61,62]
SAP130	MINCLE	Inflammatory disease and fibrosis	[30,63]
Histones	TLR2, TLR4	Sepsis, trauma, acute liver failure, kidney injury, acute lung injury	[64,65,66,67,68]
Intracellular-Cytosolic DAMPs			
ATP	P2X7, P2Y2	Sepsis, hypoxia, mechanical ventilation, migraine, stroke, cancer	[34,69,70,71]
Heat shock proteins	TLR2, TLR4, CD91, CD14, CD40	Sepsis, acute lung injury, cancer, rheumatoid arthritis, neuroinflammation	[34,72,73,74]
Uric acid	NLRP3, P2X7	Acute inflammation, ventilator-induced lung injury, kidney disease, gout	[34,75,76]
S100 proteins	TLR2, TLR4, RAGE	Endotoxin-induced shock, cancer, autoimmune disease, kidney disease, osteoarthritis	[34,77,78,79]
Galectins	CD2	Cancer, fibrosis, chronic inflammation, infection	[80,81]
CIRP	TLR-MD2	Haemorrhagic shock, sepsis	[53,82]
Amyloid-β	TLR2, NLRP1, NLRP3, RAGE	Alzheimer’s disease	[83,84]
Intracellular-Mitochondrial DAMPs			
mROS	NLRP3	Neurodegenerative disorders, cancer, pulmonary disease, cardiovascular disease, gastrointestinal disorders	[85,86]
Formyl peptides	FPR1	systemic inflammatory response syndrome, acute inflammation	[87,88]
Cytochrome C	TLR4	Neuroinflammation	[89,90]
mtDNA	TLR9	Trauma haemorrhage, sickle cell disease, arthritis, inflammation	[85,91,92,93,94]
Extracellular DAMPs			
Heparan sulfate	TLR4	Alzheimer’s disease, autoimmune disease, inflammatory disease	[95]
Fibronectin	TLR4	Diabetes, inflammation, fibrosis, liver disease, kidney disease	[96,97,98,99,100]
Fibrinogen	TLR4	Cancer, fibrosis, kidney disease	[99,101]
Hyaluronan	TLR2, TLR4, NRLP3	Fibrosis, arthritis, inflammation, kidney disease	[98,102,103]
Versican	TLR2, TLR6, CD14	Inflammatory lung diseases, cancer, kidney disease	[104]
Decorin	TLR2, TLR4	Chronic inflammation, fibrosis	[105]
Biglycan	TLR2, TLR4, NRLP3	Acute kidney injury, chronic inflammation	[106,107]
Laminin	TLR4	Autoimmune disease, fibrosis, chronic inflammatory disease, liver disease, cancer	[100,108]
Tenascin C	TLR4	Injury, fibrosis, infection, cancer	[109,110]

## Data Availability

Not applicable.
